# Weight Loss and Long-Term Weight Trajectories After a Structured Nutritional and Behavioral Intervention: A Large Real-World Cohort Study

**DOI:** 10.3390/nu18203364

**Published:** 2026-10-14

**Authors:** Odile Fabre, Sébastien Bailly, Laurent Pantagis, Rémy Legrand, Michael E. J. Lean, Luc F. Van Gaal, Susan A. Jebb, Arne Astrup

**Affiliations:** 1 Groupe Éthique et Santé, Actiburo 1, Bâtiment A, 100 Chemin de l’Aumône Vieille, 13400 Aubagne, France; laurent.pantagis@groupethiquetsante.com (L.P.); remy.legrand@groupethiquetsante.com (R.L.); 2 HP2 Laboratory, INSERM U1300, Grenoble Alpes University Hospital, University Grenoble Alpes, 38000 Grenoble, France; sbailly@chu-grenoble.fr; 3 Department of Human Nutrition, School of Medicine, Dentistry and Nursing, College of Medical, Veterinary and Life Sciences, University of Glasgow, Glasgow G31 2ER, UK; mike.lean@glasgow.ac.uk; 4 Department of Endocrinology, Diabetology and Metabolism, Antwerp University Hospital, University of Antwerp, 2650 Edegem, Belgium; luc.vangaal@uza.be; 5 Nuffield Department of Primary Care Health Sciences, University of Oxford, Oxford OX2 6GG, UK; susan.jebb@phc.ox.ac.uk; 6 Nutrition Unit, Copenhagen University Hospital—Bispebjerg and Frederiksberg, DK-2400 Copenhagen NV, Denmark; arneastrup@gmail.com

**Keywords:** obesity, overweight, weight loss, weight-loss maintenance, diet, nutritional intervention, body composition, real-world evidence

## Abstract

Background/Objectives: Achieving and maintaining intentional weight loss is a major challenge in routine clinical practice, and real-world evidence is limited. Methods: This retrospective cohort study evaluated initial weight loss in 56,093 adults from a total of 126,659 who commenced the French RNPC program between 2013 and 2025, and subsequent weight trajectories in 2395 participants with 4-year follow-up data. The intervention combined an energy-restricted, protein-enriched, low-glycemic-load diet with individualized dietary and behavioral support, followed by progressive food reintroduction and optional long-term follow-up. Results: During the weight-loss phase (median, 4.6 months), median body weight decreased by 11.0 kg (12.7%) and waist circumference by 12.0 cm (11.8%), while lean mass percentage increased by 1.3 percentage points. At 4 years, among the 2395 participants with available follow-up data, median weight remained 7.8 kg (9.1%) and waist circumference 10.0 cm (9.8%) below baseline. Longitudinal mixed-effects models using all available repeated measurements showed similar patterns in weight, BMI, waist circumference, fat mass, waist-to-height ratio, and relative fat mass at 4 years. Conclusions: These findings show substantial initial weight loss and long-term maintenance of clinically meaningful reductions in weight and waist circumference among participants with 4-year follow-up data after a structured nutritional and behavioral intervention in routine clinical practice.

## 1. Introduction

Overweight and obesity are among the leading contributors to chronic disease burden worldwide and are strongly associated with type 2 diabetes, cardiovascular diseases, metabolic dysfunction-associated steatotic liver disease (MASLD), obstructive sleep apnea, musculoskeletal disorders, and reduced quality of life [1]. Obesity is now widely recognized as a chronic, relapsing, multifactorial disease requiring long-term management strategies rather than short-term interventions [1,2].

Lifestyle-based interventions remain the cornerstone of obesity management and are recommended as first-line therapy by major scientific societies and public health agencies, although long-term maintenance of weight loss remains particularly challenging in real-world settings [1,2,3,4]. Most studies have shown that a substantial proportion of individuals progressively regain weight after the end of structured interventions, with recent systematic evidence confirming that the effects of behavioral weight-management interventions tend to attenuate over time [5]. This progressive weight regain reflects multiple biological, behavioral, and environmental mechanisms [6]. Weight regain usually begins within the first year after intervention discontinuation despite initial clinically significant weight reduction [7,8,9].

While randomized clinical trials provide important efficacy data, they often include highly selected populations, intensive monitoring conditions, and relatively short follow-up periods, therefore limiting their applicability to routine clinical practice [10]. Long-term real-world observational studies can complement randomized trials by characterizing long-term outcomes associated with lifestyle-based obesity interventions in large and heterogeneous populations under routine clinical conditions. This issue is particularly relevant for multidisciplinary programs integrating nutritional counseling, behavioral support and prolonged follow-up, which may offer more durable outcomes than isolated dietary interventions.

The nutritional strategy implemented in the French Rééducation Nutritionnelle et Psycho-Comportementale (RNPC; Nutritional Psycho-Behavioral Re-education) program is partly based on the concepts evaluated in the European DiOGenes project [11]. In that trial, a moderately high-protein (25% energy), low-glycemic-index diet was associated with better long-term weight-loss maintenance after an 11 kg initial weight reduction. In the intention-to-treat analysis, weight regain was approximately 1 kg lower with high-protein than with low-protein diets and approximately 1 kg lower with low- than with high-glycemic-index diets. Among participants who completed the intervention, only the low-protein/high-glycemic-index diet was associated with significant weight regain (+1.67 kg) during the 6-month follow-up [11]. The RNPC program represents a real-world clinical application of these principles through a structured multidisciplinary intervention combining a protein-enriched, low-glycemic-load nutritional approach with psycho-behavioral support and prolonged follow-up.

Since 2018, studies conducted within the RNPC research network have examined weight-loss outcomes among participants in the program. The initial retrospective RNPC cohort study evaluated weight loss and weight-loss maintenance for up to 36 weeks among program completers [12]. Subsequent research also suggested the potential importance of continued dietary follow-up, with greater weight loss observed among participants who maintained remote consultations during the COVID-19 lockdown [13]. Some participants included in the present analysis also contributed data to these previous RNPC publications, as the studies drew, at least in part, on the same underlying clinical database. However, the present study is a distinct analysis based on an extended recruitment period, a substantially larger analytical cohort, and longer follow-up than previous RNPC studies.

Available long-term studies suggest that although partial weight regain is common, most participants remain below their baseline weight several years after intervention. In addition to the DiOGenes study, participants in the Look AHEAD trial maintained an average weight loss of approximately 6% after 8 years [4]. Long-term weight loss has also been reported in routine clinical practice: in a retrospective cohort of 10,693 participants enrolled in a medically supervised weight-management program, mean weight remained 6.4 kg below baseline at 5 years among participants with available follow-up data [14]. However, large real-world evaluations of multidisciplinary nutritional and psycho-behavioral programs remain scarce. Long-term evaluation is essential because the durability of behavioral changes ultimately determines the clinical value of non-pharmacological interventions. Importantly, the growing use of highly effective anti-obesity medications, including GLP-1 receptor agonists (GLP-1 RAs), has renewed interest in identifying complementary lifestyle approaches capable of sustaining long-term benefits after medication discontinuation or beyond the actively supported intervention phase [15].

This study aimed to describe changes in body weight, waist circumference, and body composition during the weight-loss phase and subsequent long-term weight trajectories up to 4 years among adults participating in the RNPC program in routine clinical practice.

## 2. Materials and Methods

### 2.1. Study Design

This retrospective observational cohort study analyzed routinely collected, anonymized data from adults participating in the RNPC program. The study complied with the Declaration of Helsinki and applicable French regulations governing observational research using anonymized health data. Under French regulations, ethics committee approval and written informed consent were not required for this type of study. Participants were informed that their anonymized data could be used for research purposes and could object at any time. This study followed the STROBE reporting guideline.

### 2.2. Participants

Adults with a first measurement recorded in the RNPC database between 1 January 2013 and 31 December 2025 were considered for inclusion. Among them, 126,659 participants started the RNPC program with the weight-loss phase. For inclusion in the present analysis, participants were required to meet the anthropometric eligibility criteria, have at least three measurements during the first 90 days of the program, and have a measurement documenting entry into the weight-maintenance phase. The final study population therefore comprised 56,093 participants with documented completion of the weight-loss phase (Figure S1). Exclusion criteria were age < 18 years, body mass index (BMI) < 25 kg/m^2^, waist circumference < 80 cm for females and <94 cm for males, pregnancy, and breastfeeding. Regarding medical exclusion criteria, individuals considered clinically frail based on an overall assessment taking into account factors such as age over 80 years, heart, liver, or kidney failure, active cancer, multiple and/or severe comorbidities, and polypharmacy were excluded from the study because they started the program directly by the weight maintenance phase, in order to avoid risks associated with severe energy restriction. These factors were considered collectively and did not constitute independent exclusion criteria. Individuals with type 1 diabetes and type 2 diabetes with insulin therapy were allowed to follow the program but only with formal agreement of their attending physician. The use of anti-obesity medications, identified from prescriptions for Saxenda (liraglutide), Wegovy (semaglutide), and Mounjaro (tirzepatide), was recorded.

Race and ethnicity were not included because these data are not routinely collected in the RNPC database. Under French regulations, the collection of race- and ethnicity-related data is strictly regulated and is generally not undertaken in routine clinical practice; consequently, these variables were unavailable for this retrospective study.

### 2.3. Weight-Loss and Maintenance Program

The RNPC program has previously been described in detail [12]. Nutritional counseling and follow-up consultations were included in the cost of the nutritional products supplied throughout the program. The RNPC program was entirely funded by participants and received no reimbursement from the French healthcare system.

The RNPC program is organized into two successive phases. The initial weight-loss phase aimed to achieve an individually defined target weight. This phase was based on a hypocaloric diet providing approximately 800–1000 kcal/day. Protein intake was individualized according to current body weight and sex, with target intakes of approximately 1.2 g protein/kg/day in females and 1.5 g protein/kg/day in males. Foods with a high-glycemic load were limited. Actual dietary intake was not systematically assessed using food diaries or other standardized dietary assessment tools; therefore, quantitative data on adherence to the prescribed energy and protein intakes were not available.

Participants also attended face-to-face consultations every two weeks with a trained dietitian, allowing individualized counseling, anthropometric assessments, and behavioral support. The duration of this phase varies according to individual weight-loss goals and participant progress.

Before initiation of the weight-loss phase, a biological assessment was requested, with serum creatinine measurement required to assess renal function. Estimated glomerular filtration rate (eGFR) was calculated using the MDRD equation, and prescribed protein intake was adapted according to renal function in accordance with French Haute Autorité de Santé recommendations [16]. A repeat biological assessment was strongly recommended, although not mandatory, after participants reached the weight-maintenance phase. During the program, dietitians could adapt the dietary prescription according to eGFR, recommend discontinuation of the intervention, or refer participants to their treating physician when a health issue required medical evaluation beyond their scope of practice. Adverse events and reasons for treatment discontinuation, when known, were recorded in individual medical records but were not available for analysis in the present study.

The maintenance phase began once the target weight had been reached. The first step consisted of gradually reintroducing carbohydrates and dietary fats over a period of at least 3 months, depending on the extent of weight loss, while maintaining weight stability. During this period, intensive follow-up (every two weeks) was maintained.

The second step of the maintenance phase was intended to promote long-term adherence to healthy eating habits, physical activity, and behavioral changes. At this stage, no structured follow-up was mandated. However, participants could continue attending follow-up visits voluntarily and at no additional cost, generating the long-term observational data analyzed in the present study.

The core structure and nutritional principles of the RNPC program remained consistent throughout the study period and were standardized across participating centers. Although minor operational adjustments occurred between 2013 and 2025, the main components of the intervention—including the energy-restricted, protein-enriched diet, fortnightly dietitian consultations, behavioral support, and progressive food reintroduction—remained unchanged.

### 2.4. Anthropometric Measurements

Body weight and body composition were assessed at baseline and at each follow-up visit by trained dietitians using calibrated bioelectrical impedance analyzers. Measurements were performed during routine clinical visits and were not systematically standardized with respect to fasting status, hydration status, or time of day. Fat mass and lean mass were defined in the present study as the fat and lean mass percentages estimated by the bioelectrical impedance analyzer and expressed as a percentage of total body weight. Absolute fat and lean mass in kilograms were not available in the database and could therefore not be analyzed. Because the study was conducted across multiple centers, several bioelectrical impedance scale models were used, and fat and lean mass values therefore represent a device-derived BIA estimate rather than a direct measurement of fat or fat-free mass. For each participant, longitudinal measurements were obtained using the same bioelectrical impedance analyzer model throughout follow-up, thereby limiting the impact of inter-device variability on within-participant changes.

Waist circumference was measured midway between the lower costal margin and the iliac crest, typically 2–5 cm above the umbilicus.

Waist-to-height ratio and relative fat mass were also calculated, providing complementary information on fat distribution and body fatness [17,18].

### 2.5. Statistical Analyses

Continuous variables are described as median and interquartile range (IQR), and categorical variables as numbers and percentages. Baseline characteristics were compared between males and females using Wilcoxon rank-sum tests for continuous variables and Chi-square or Fisher’s exact tests, as appropriate, for categorical variables.

Absolute and relative changes in anthropometric parameters were calculated at the individual level between predefined time points. For each parameter, descriptive analyses were restricted to participants with available measurements at both time points being compared. Thus, the number of participants contributing to each descriptive comparison varied according to data availability at the corresponding time points. Changes were summarized as median (IQR) for the overall population and separately for males and females. The magnitude of absolute change was additionally summarized using the standardized response mean (SRM), calculated as the absolute mean individual change divided by the standard deviation of individual changes. SRMs were interpreted as negligible (<0.20), small (0.20–0.49), moderate (0.50–0.79), or large (≥0.80).

Long-term follow-up time points were defined relative to the end of the weight-loss phase. Measurements at 1, 2, 3, and 4 years were selected within prespecified windows of 274–548, 639–1095, 1004–1460, and 1369–1825 days after the end of the weight-loss phase, respectively. At each time point, only the measurement closest to the target date (days 365, 730, 1095, and 1460, respectively) was retained per participant; overlapping windows were handled sequentially to prevent reuse of the same measurement.

In secondary analyses, adjusted longitudinal changes were assessed using separate linear mixed-effects models for each anthropometric parameter. The outcome was the individual absolute change from baseline at each available post-baseline time point (end of the weight-loss phase [EWL] and years 1–4); baseline itself was not entered as an outcome observation. Time was represented by categorical indicators for years 1–4, with EWL as the reference. Descriptive summaries at each time point included all participants with an available measurement of the relevant anthropometric variable at that time point. Models included a patient-level random intercept to account for within-patient correlation; no additional residual covariance structure was specified, and they were adjusted for baseline age, sex, and the baseline value of the anthropometric parameter. Continuous covariates were centered on their means in the parameter-specific analytical sample. Models were fitted by restricted maximum likelihood. Participants with a baseline measurement and at least one post-baseline measurement contributed all available post-baseline measurements through year 4; complete data at all follow-up time points were not required. Missing follow-up measurements were not imputed; estimates from the mixed-effects models rely on a missing-at-random assumption conditional on the variables and observed outcomes included in the model. Adjusted mean changes and 95% confidence intervals (CIs) were estimated for baseline to the end of the weight-loss phase, for the end of the weight-loss phase to 4 years, and for baseline to 4 years using, respectively, the model intercept, the year-4 coefficient, and their sum at the mean covariate values of the analytical sample.

To assess the potential impact of selective availability of four-year measurements, we performed a sensitivity analysis using inverse probability of observation weighting (see Supplementary Methods). The analysis included participants with an opportunity to enter the predefined year-4 measurement window before the administrative data cutoff. The probability of year-4 BMI availability was estimated from baseline, end-of-weight-loss, follow-up availability, and calendar-time characteristics. Stabilized inverse probability of observation weights was used to estimate mean BMI changes from baseline to year 4, with weights truncated at the 1st and 99th percentiles in the primary analysis and untruncated weights examined in a sensitivity analysis.

Statistical analyses were performed using SAS software, version 9.4 (SAS Institute Inc., Cary, NC, USA), and figures were generated using R version 4.5.1 (R Core Team, R Foundation for Statistical Computing, Vienna, Austria). A two-sided *p* value < 0.05 was considered statistically significant.

## 3. Results

### 3.1. Study Population

A total of 56,093 participants met the analytical inclusion criteria and had documented completion of the weight-loss phase, and were therefore included (Table 1, Figure S1). At baseline, the study population was predominantly female (75.9%) and nearly two-thirds (66.5%) had a BMI ≥30 kg/m^2^. Baseline characteristics differed substantially between males and females. Males were slightly older and had higher body weight, waist circumference, BMI, lean mass percentage and body water percentage, whereas females had higher fat mass percentage. Sleep apnea, type 2 diabetes, dyslipidemia and MASH were more frequent in males, while osteoarthritis, smoking, pain, dyspnea, stress and sleep complaints were more frequent in females. Most between-sex differences were statistically significant, except sedentary lifestyle, which was similarly distributed in both groups (Table 1). Compared with non-included participants (*N* = 63,145), those included in the longitudinal study had lower baseline adiposity, reflected by lower BMI, body weight, obesity prevalence, waist circumference, waist-to-height ratio, fat mass, and relative fat mass, and were marginally older. Absolute SMDs exceeded 0.10 for several baseline characteristics, with the largest differences observed for BMI (0.207), body weight (0.171), obesity prevalence (0.156), and body water percentage (0.151) (Table S1).

Recruitment increased over the study period, from 1355 participants entering the program in 2013 to 7353 in 2024 (Table S2). Of the 56,093 participants who completed the weight-loss phase, 27,391 (48.8%) had reached the nominal 4-year date (day 1460 after the end of the weight-loss phase) by 31 December 2025.

### 3.2. Changes in Anthropometric Characteristics During the Weight-Loss Phase

The median duration of the weight-loss phase was 4.6 months (IQR, 3.2; 6.7), 3.9 months (IQR, 2.7; 5.9) in males and 4.8 months (IQR, 3.4; 6.9) in females.

Among the 56,093 participants who completed the weight-loss phase, 2733 (4.9%) lost 0 to <5% of their initial body weight, 13,802 (24.6%) lost 5 to <10%, 20,301 (36.2%) lost 10 to <15%, and 19,082 (34.0%) lost ≥15%, whereas 175 (0.3%) gained weight.

From baseline to the end of the weight-loss phase, median body weight decreased by 12.7% (IQR, −16.7; −9.3), waist circumference by 11.8% (IQR, −15.4; −8.6), fat mass by 10.3% (IQR, −15.4; −6.3), waist-to-height ratio by 11.8% (IQR, −15.4; −8.6), and relative fat mass by 10.2% (IQR, −13.9; −7.1), whereas lean mass percentage increased by 1.3 percentage points, corresponding to a 4.5% relative increase (IQR, +2.1; +8.1) (Table 2 and Table S3, Figure S2).

Among participants with BMI measured at both time points (*N* = 56,093), 40,762 (72.7%) moved to a lower BMI category, 15,311 (27.3%) remained unchanged, and only 20 (<0.1%) moved to a higher category. Overall, 58.2% of participants who were overweight at baseline reached the normal-BMI range. Among participants with obesity, 83.9% with baseline BMI 30 to <35 kg/m^2^ and 74.7% with BMI ≥35 kg/m^2^ moved to a lower BMI category. Consequently, the proportion of participants with obesity decreased from 66.5% at baseline to 28.6% at the end of the weight-loss phase, and 22.3% of the overall population reached the normal-BMI range (Figure S3 and Table S4).

Changes were similar in both sexes, although absolute reductions in weight and waist circumference were greater in males. Standardized response means indicated large magnitudes of change for most adiposity-related outcomes.

Adjusted mixed-effects models confirmed these findings after adjustment for age, sex, and baseline anthropometric values (all *p* < 0.001; Table S5).

### 3.3. Long-Term Changes in Anthropometric Characteristics

Among the 56,093 participants who completed the weight-loss phase, 54,361 (96.9%) had at least one recorded measurement during the subsequent structured weight-maintenance period, with a median of six measurement dates (IQR, 4; 9) over a median interval of 3.1 months (IQR, 1.9; 5.4) between the first and last measurements during this period. A total of 32,870 participants (58.6%) had at least one measurement during the subsequent long-term follow-up, with a median of four measurement dates (IQR, 2; 8) spanning a median interval of 4.9 months (IQR, 0.9; 13.9).

Among the 27,391 participants who had reached the nominal 4-year date, 2317 (8.5%) had a measurement within the prespecified year-4 window, corresponding to 4.1% of the full cohort. An additional 78 participants had a year-4-window measurement before reaching day 1460, yielding 2395 participants (4.3% of the full cohort) with a year-4 measurement overall.

After the weight-loss phase, anthropometric data were available for 22,601 participants at 1 year, 9738 at 2 years, 4684 at 3 years, and 2395 at 4 years of follow-up (Table 2, Figure S1). Participants with and without 4-year follow-up were broadly comparable at baseline, although those followed at 4 years had a slightly lower baseline weight. In contrast, at the end of the weight-loss phase, participants subsequently retained at 4 years had lower weight, BMI, waist circumference and waist-to-height ratio, and had achieved greater initial reductions in weight and waist circumference, suggesting selective long-term follow-up among participants with a stronger initial response (Table S6).

**Figure 1:**
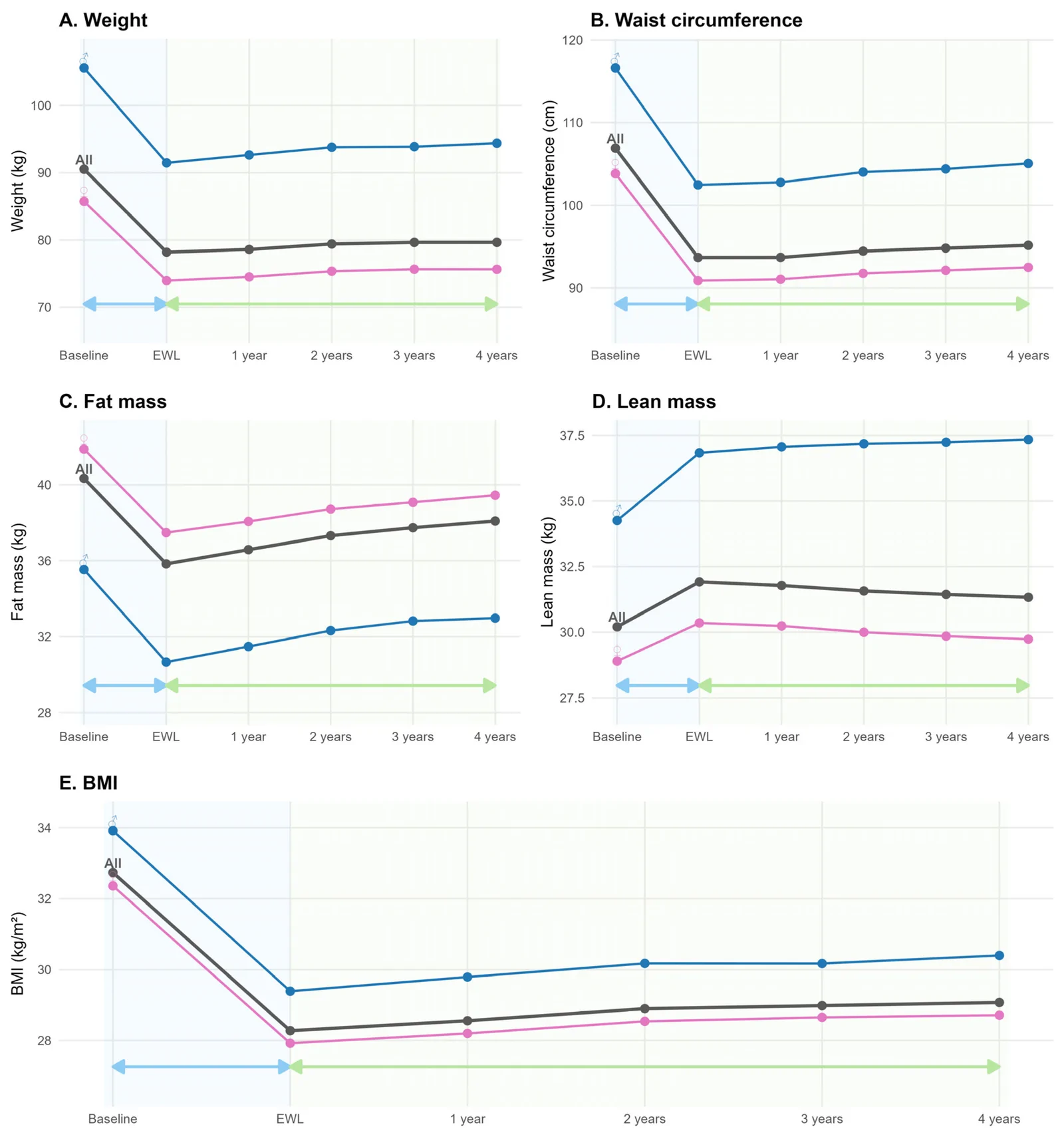
Long-term changes in anthropometric characteristics during follow-up. In each panel, the three lines represent the median values for the overall population (black line), males (blue line), and females (pink line). Values at each time point represent cross-sectional summaries of participants with available data at that assessment; therefore, the connected lines do not represent trajectories of a consistently followed cohort. (**A**) For body weight, the numbers of participants with available data were 56,093 at baseline, 56,093 at the end of the weight-loss phase, 22,601 at 1 year, 9738 at 2 years, 4684 at 3 years, and 2395 at 4 years. (**B**) For waist circumference, the numbers of participants with available data were 56,076 at baseline, 56,037 at the end of the weight-loss phase, 22,556 at 1 year, 9722 at 2 years, 4677 at 3 years, and 2394 at 4 years. (**C**) For fat mass percentage, the numbers of participants with available data were 53,575 at baseline, 50,962 at the end of the weight-loss phase, 19,937 at 1 year, 8389 at 2 years, 4010 at 3 years, and 2005 at 4 years. (**D**) For lean mass percentage, the numbers of participants with available data were 53,540 at baseline, 50,944 at the end of the weight-loss phase, 19,919 at 1 year, 8376 at 2 years, 4008 at 3 years, and 2002 at 4 years. (**E**) For BMI, the numbers of participants with available data were 56,093 at baseline, 56,093 at the end of the weight-loss phase, 22,601 at 1 year, 9738 at 2 years, 4684 at 3 years, and 2395 at 4 years.

Among participants with measurements through 4 years, partial regain occurred after the end of the weight-loss phase. Median weight increased by 4.7 kg (IQR, 0.9; 8.8), corresponding to 6.5% (IQR, 1.3; 12.1), and waist circumference increased by 4.0 cm (IQR, −1.0; 9.0), corresponding to 3.9% (IQR, −1.1; 9.4). Nevertheless, substantial reductions from baseline were still observed: median weight was reduced by 7.8 kg (IQR, −13.1; −3.7), or 9.1% (IQR, −14.5; −4.4), and waist circumference by 10.0 cm (IQR, −16.0; −5.0), or 9.8% (IQR, −15.1; −4.9). Waist-to-height ratio and relative fat mass also remained below baseline, whereas lean mass percentage remained stable (Table S7, Figure 1).

Longitudinal mixed-effects models using all available repeated measurements showed similar patterns. From the end of the weight-loss phase to 4-year follow-up, weight increased by 5.4 kg (95% CI, 5.2 to 5.5) and waist circumference by 4.0 cm (95% CI, 3.7 to 4.2). However, at 4-year follow-up, adjusted changes from baseline were −7.0 kg (95% CI, −7.1 to −6.8) for weight and −9.3 cm (95% CI, −9.5 to −9.0) for waist circumference. BMI, fat mass, waist-to-height ratio, and relative fat mass also remained improved compared with baseline, while lean mass percentage remained slightly higher, by 0.9 percentage points (95% CI, 0.8 to 1.1), all with *p* < 0.001 (Table S5).

In the sensitivity analysis addressing selective availability of 4-year measurements, 2395 of 29,190 participants eligible to enter the year-4 measurement window (8.2%) had an observed year-4 BMI. Among those observed, the mean BMI change from baseline was −3.25 kg/m^2^ without weighting. After inverse probability of observation weighting, the estimated mean change was −1.92 kg/m^2^ with weights truncated at the 1st and 99th percentiles, and −1.89 kg/m^2^ without truncation (Table S8).

## 4. Discussion

In this large retrospective real-world cohort, marked and clinically meaningful weight loss was observed among adults with excess weight and increased waist circumference who completed the weight-loss phase of the RNPC program. This study also provides important data on subsequent long-term weight trajectories after discontinuation of intensive follow-up (i.e., every two weeks). Although partial weight regain occurred, participants with available 4-year follow-up data maintained a substantial proportion of their initial weight loss four years after completion of the weight-loss phase.

Weight-loss maintenance cannot rely solely on transient caloric restriction but requires sustained behavioral changes including healthier eating habits, regular physical activity, routine self-weighing, and coping strategies. Previous findings from the National Weight Control Registry support the importance of these long-term behavioral adaptations [19]. Accordingly, the RNPC program was designed to promote progressive nutritional re-education and psycho-behavioral changes beyond the active weight-loss phase.

Previous studies have suggested that structured and supervised lifestyle interventions generally produce more durable outcomes than isolated or poorly supervised dietary approaches [4,6]. The multidisciplinary structure of the RNPC program represents one potential explanation for the observed outcomes, although the present study was not designed to determine the respective contributions of its individual components. Repeated interactions with trained dietitians, behavioral reinforcement, progressive dietary stabilization, and individualized counseling were intended to improve adherence and facilitate the integration of healthier lifestyle habits over time.

Nevertheless, maintaining long-term follow-up in routine clinical practice is challenging. The proportion of participants with available data decreased progressively over time, with only a relatively small proportion of the initial cohort providing data for evaluation four years after completion of the weight-loss phase. Attrition is common in long-term obesity studies conducted under real-life conditions and reflects the inherent difficulty of maintaining prolonged patient engagement outside controlled research settings [10]. This selective retention may have resulted in an overestimation of long-term weight loss. Indeed, those retained at 4 years had achieved greater initial reductions in weight and waist circumference. Among participants eligible to enter the year-4 measurement window, only 8.2% had an observed year-4 BMI. In the sensitivity analysis accounting for observed predictors of follow-up availability using inverse probability of observation weighting, the estimated mean BMI reduction from baseline to 4 years was attenuated from 3.25 kg/m^2^ in the unweighted analysis to 1.92 kg/m^2^ after weighting. Importantly, BMI remained below baseline after weighting. However, residual selection bias related to unmeasured factors cannot be excluded, and the reported confidence intervals reflect uncertainty under the fitted models but do not capture such potential bias due to attrition. Moreover, because long-term consultations were voluntary, participants with available follow-up measurements may also have differed in their level of continued engagement with the RNPC program, and the potential contribution of ongoing professional support to the observed long-term weight trajectories cannot be disentangled from weight maintenance occurring independently of such support. Nevertheless, such studies provide valuable information on long-term weight trajectories among participants with available follow-up data in routine practice.

Another consideration relates to the potential influence of recently introduced anti-obesity pharmacotherapies. During the study period, Saxenda (liraglutide), Wegovy (semaglutide), and Mounjaro (tirzepatide) progressively became available in France. However, exposure to these treatments within the present cohort was extremely limited. Overall, only nine participants (0.02% at baseline, Table 1) received one of these medications, including just four treated with Saxenda, the only GLP-1 RA available early enough to potentially influence 4-year outcomes. Exposure to Wegovy and Mounjaro was negligible because these agents became available only at the end of, or after, the inclusion period.

Adequate protein intake and regular physical activity have been emphasized in recent expert recommendations as important supportive measures during GLP-1 RA treatment, particularly to help preserve lean body mass [20,21,22]. The nutritional and behavioral principles of the RNPC program are consistent with these recommendations. Given the chronic nature of obesity and the frequent weight regain observed after discontinuation of anti-obesity medications [23,24], structured lifestyle interventions may have a complementary role alongside pharmacotherapy. The potential added value of combining the RNPC program with contemporary anti-obesity medications therefore warrants evaluation in prospective studies.

This study has several limitations. Its retrospective, uncontrolled design precludes causal attribution of the observed changes to the RNPC intervention and does not allow direct comparison with usual care or alternative weight-management strategies. In addition, the study population was restricted to participants with documented completion of the weight-loss phase; therefore, the observed weight-loss outcomes may not be representative of all individuals who initiated the RNPC program. Body composition was estimated using commercial bioimpedance analyzers, with several device models used across centers and without systematic standardization of fasting status, hydration, or time of measurement, limiting the precision and comparability of longitudinal body-composition estimates. Although adverse events and reasons for discontinuation were recorded in individual medical records when known, these data were not available for analysis; therefore, the present study was not designed to assess the safety or tolerability of the intervention. Nevertheless, the large initial cohort, availability of repeated measurements in routine clinical practice, and consistency of findings across anthropometric outcomes provide valuable real-world evidence on long-term weight trajectories after a structured nutritional and behavioral intervention.

## 5. Conclusions

In this large real-world cohort of participants who completed the RNPC weight-loss phase, substantial reductions in body weight and abdominal adiposity were observed during this first stage of the program. Among participants with available long-term follow-up, clinically meaningful reductions in body weight and waist circumference were maintained for up to 4 years despite partial weight regain. These findings characterize weight trajectories among RNPC participants with available follow-up data and provide a rationale for prospective controlled studies assessing long-term outcomes, including when such programs are combined with contemporary anti-obesity pharmacotherapies.

## Figures and Tables

**Table 1:** Participant characteristics at baseline.

Characteristic	All (*N* = 56,093)	Males (*N* = 13,537; 24.1%)	Females (*N* = 42,556; 75.9%)	Missing	*p* Value
Age, y	54.0 (45.0; 63.0)	56.0 (47.0; 64.0)	54.0 (45.0; 63.0)	0	<0.001
BMI categories, *N* (%)				0	<0.001
25 to <30 kg/m^2^	18,828 (33.6%)	2869 (21.2%)	15,959 (37.5%)		
30 to <35 kg/m^2^	21,191 (37.8%)	5793 (42.8%)	15,398 (36.2%)		
≥35 kg/m^2^	16,074 (28.7%)	4875 (36.0%)	11,199 (26.3%)		
Current smoker, *N* (%)	5577 (9.9%)	1227 (9.1%)	4350 (10.2%)	0	<0.001
Menopause, *N* (%)	22,994 (41.0%)	NA	22,994 (54.0%)	0	NA
Sleep apnea, *N* (%)	9414 (16.8%)	4392 (32.4%)	5022 (11.8%)	0	<0.001
Osteoarthritis, *N* (%)	18,260 (32.6%)	3757 (27.8%)	14,503 (34.1%)	0	<0.001
Sedentary lifestyle, *N* (%)	18,417 (32.8%)	4388 (32.4%)	14,029 (33.0%)	0	0.234
MASH, *N* (%)	2548 (4.5%)	1024 (7.6%)	1524 (3.6%)	0	<0.001
Diabetes, *N* (%)	5102 (9.6%)	2275 (17.8%)	2827 (7.0%)	3063	<0.001
Dyslipidemia, *N* (%)	42,745 (78.7%)	10,315 (78.5%)	32,430 (78.8%)	1795	0.575
GLP-1 receptor agonist treatment, *N* (%)	9 (0.02%)	2 (0.01%)	7 (0.02%)	0	1.000

Values are median (interquartile range) or number (*N*) and percentage of participants (%). *p* values are for the comparison between males and females using Wilcoxon rank-sum tests for continuous variables and Chi-square or Fisher’s exact tests for categorical variables. BMI, body mass index; MASH, metabolic dysfunction-associated steatohepatitis; NA, not applicable.

**Table 2:** Description of anthropometric characteristics at each time point.

Parameter	Group	Statistic	Baseline	End of Weight-Loss Phase	1-Year Follow-Up	2-Year Follow-Up	3-Year Follow-Up	4-Year Follow-Up
Weight, kg	All	Median (IQR)	87.90 (77.60; 100.40)	75.70 (67.40; 86.70)	76.00 (67.60; 87.20)	76.80 (68.40; 88.10)	77.10 (68.40; 88.30)	77.00 (68.80; 87.90)
		*N* (%)	56,093 (100.0%)	56,093 (100.0%)	22,601 (40.3%)	9738 (17.4%)	4684 (8.4%)	2395 (4.3%)
	Males	Median (IQR)	103.20 (93.70; 115.20)	89.70 (82.10; 98.50)	90.80 (82.60; 100.50)	92.00 (83.60; 101.80)	91.80 (83.80; 101.90)	92.40 (83.40; 102.20)
		*N* (%)	13,537 (100.0%)	13,537 (100.0%)	5089 (37.6%)	2140 (15.8%)	1025 (7.6%)	511 (3.8%)
	Females	Median (IQR)	83.50 (75.10; 94.00)	71.70 (65.40; 80.30)	72.40 (65.70; 81.30)	73.30 (66.50; 82.00)	73.50 (66.60; 82.60)	73.40 (66.60; 82.05)
		*N* (%)	42,556 (100.0%)	42,556 (100.0%)	17,512 (41.2%)	7598 (17.9%)	3659 (8.6%)	1884 (4.4%)
BMI, kg/m^2^	All	Median (IQR)	31.90 (28.90; 35.60)	27.50 (25.20; 30.50)	27.80 (25.40; 30.90)	28.20 (25.60; 31.30)	28.20 (25.70; 31.40)	28.30 (25.70; 31.40)
		*N* (%)	56,093 (100.0%)	56,093 (100.0%)	22,601 (40.3%)	9738 (17.4%)	4684 (8.4%)	2395 (4.3%)
	Males	Median (IQR)	33.20 (30.40; 36.60)	28.80 (26.70; 31.40)	29.20 (26.90; 31.90)	29.50 (27.20; 32.30)	29.50 (27.20; 32.30)	29.70 (27.20; 32.70)
		*N* (%)	13,537 (100.0%)	13,537 (100.0%)	5089 (37.6%)	2140 (15.8%)	1025 (7.6%)	511 (3.8%)
	Females	Median (IQR)	31.40 (28.50; 35.20)	27.00 (24.80; 30.10)	27.30 (25.00; 30.50)	27.70 (25.30; 30.80)	27.70 (25.40; 31.00)	27.90 (25.50; 31.10)
		*N* (%)	42,556 (100.0%)	42,556 (100.0%)	17,512 (41.2%)	7598 (17.9%)	3659 (8.6%)	1884 (4.4%)
Waist circumference, cm	All	Median (IQR)	106.00 (97.00; 115.00)	93.00 (85.00; 101.00)	92.00 (85.00; 101.00)	93.00 (85.00; 102.00)	94.00 (85.00; 103.00)	94.00 (86.00; 103.00)
		*N* (%)	56,076 (100.0%)	56,037 (99.9%)	22,556 (40.2%)	9722 (17.3%)	4677 (8.3%)	2394 (4.3%)
	Males	Median (IQR)	115.00 (108.00; 124.00)	101.00 (95.00; 109.00)	102.00 (95.00; 110.00)	103.00 (96.00; 111.00)	103.00 (96.00; 112.00)	104.00 (97.00; 112.00)
		*N* (%)	13,530 (99.9%)	13,521 (99.9%)	5080 (37.5%)	2137 (15.8%)	1023 (7.6%)	510 (3.8%)
	Females	Median (IQR)	103.00 (95.00; 111.00)	90.00 (83.00; 97.00)	90.00 (83.00; 98.00)	90.00 (84.00; 99.00)	91.00 (84.00; 99.00)	91.00 (84.00; 100.00)
		*N* (%)	42,546 (100.0%)	42,516 (99.9%)	17,476 (41.1%)	7585 (17.8%)	3654 (8.6%)	1884 (4.4%)
Fat mass, %	All	Median (IQR)	40.40 (36.60; 44.30)	36.10 (32.10; 40.00)	36.70 (32.80; 40.70)	37.40 (33.50; 41.40)	37.90 (34.10; 41.60)	38.30 (34.40; 42.00)
		*N* (%)	53,575 (95.5%)	50,962 (90.9%)	19,937 (35.5%)	8389 (15.0%)	4010 (7.1%)	2005 (3.6%)
	Males	Median (IQR)	35.75 (31.40; 39.70)	31.10 (26.30; 35.20)	31.90 (27.20; 35.90)	32.45 (28.10; 36.60)	33.10 (28.30; 37.10)	32.90 (29.10; 37.30)
		*N* (%)	13,000 (96.0%)	12,321 (91.0%)	4507 (33.3%)	1838 (13.6%)	860 (6.4%)	421 (3.1%)
	Females	Median (IQR)	41.60 (38.30; 45.30)	37.30 (33.90; 41.00)	37.90 (34.40; 41.60)	38.50 (35.10; 42.20)	39.00 (35.50; 42.50)	39.40 (36.00; 42.60)
		*N* (%)	40,575 (95.3%)	38,641 (90.8%)	15,430 (36.3%)	6551 (15.4%)	3150 (7.4%)	1584 (3.7%)
Lean mass, %	All	Median (IQR)	29.20 (26.90; 32.40)	30.80 (28.20; 34.70)	30.30 (28.00; 34.30)	30.00 (27.80; 33.90)	29.80 (27.50; 33.80)	29.50 (27.50; 33.30)
		*N* (%)	53,540 (95.4%)	50,944 (90.8%)	19,919 (35.5%)	8376 (14.9%)	4008 (7.1%)	2002 (3.6%)
	Males	Median (IQR)	34.00 (31.00; 36.30)	36.30 (33.70; 38.70)	36.10 (33.40; 38.50)	35.80 (33.10; 38.30)	35.70 (32.90; 38.10)	35.60 (32.60; 38.10)
		*N* (%)	12,979 (95.9%)	12,313 (91.0%)	4501 (33.2%)	1834 (13.5%)	861 (6.4%)	419 (3.1%)
	Females	Median (IQR)	28.40 (26.40; 30.50)	29.70 (27.70; 32.00)	29.40 (27.50; 31.70)	29.15 (27.20; 31.40)	29.00 (27.00; 31.00)	28.70 (26.90; 30.90)
		*N* (%)	40,561 (95.3%)	38,631 (90.8%)	15,418 (36.2%)	6542 (15.4%)	3147 (7.4%)	1583 (3.7%)
Body water, %	All	Median (IQR)	42.90 (39.60; 45.70)	46.20 (43.00; 49.10)	45.70 (42.60; 48.60)	45.20 (42.00; 48.00)	44.90 (41.70; 47.70)	44.50 (41.55; 47.40)
		*N* (%)	53,344 (95.1%)	50,838 (90.6%)	19,901 (35.5%)	8372 (14.9%)	4006 (7.1%)	2000 (3.6%)
	Males	Median (IQR)	45.80 (42.70; 48.60)	49.10 (46.20; 52.10)	48.70 (45.90; 51.50)	48.10 (45.50; 51.00)	48.00 (45.00; 50.60)	47.90 (44.60; 50.30)
		*N* (%)	12,933 (95.5%)	12,290 (90.8%)	4499 (33.2%)	1832 (13.5%)	858 (6.3%)	419 (3.1%)
	Females	Median (IQR)	42.10 (39.00; 44.70)	45.30 (42.30; 48.00)	44.90 (41.90; 47.50)	44.40 (41.40; 47.10)	44.10 (41.10; 46.80)	43.80 (41.00; 46.40)
		*N* (%)	40,411 (95.0%)	38,548 (90.6%)	15,402 (36.2%)	6540 (15.4%)	3148 (7.4%)	1581 (3.7%)
Waist-to-height ratio	All	Median (IQR)	0.64 (0.59; 0.69)	0.56 (0.52; 0.61)	0.56 (0.51; 0.61)	0.56 (0.52; 0.61)	0.57 (0.52; 0.62)	0.57 (0.52; 0.62)
		*N* (%)	56,076 (100.0%)	56,037 (99.9%)	22,556 (40.2%)	9722 (17.3%)	4677 (8.3%)	2394 (4.3%)
	Males	Median (IQR)	0.66 (0.61; 0.71)	0.57 (0.54; 0.62)	0.58 (0.54; 0.62)	0.58 (0.54; 0.63)	0.58 (0.55; 0.63)	0.59 (0.55; 0.64)
		*N* (%)	13,530 (99.9%)	13,521 (99.9%)	5080 (37.5%)	2137 (15.8%)	1023 (7.6%)	510 (3.8%)
	Females	Median (IQR)	0.63 (0.58; 0.69)	0.55 (0.51; 0.60)	0.55 (0.51; 0.60)	0.56 (0.51; 0.61)	0.56 (0.51; 0.61)	0.56 (0.52; 0.62)
		*N* (%)	42,546 (100.0%)	42,516 (99.9%)	17,476 (41.1%)	7585 (17.8%)	3654 (8.6%)	1884 (4.4%)
**Relative fat mass, %**	All	Median (IQR)	42.67 (37.63; 46.00)	37.90 (32.93; 41.65)	38.05 (33.18; 41.96)	38.53 (33.69; 42.22)	38.62 (33.66; 42.38)	38.82 (34.02; 42.60)
		*N* (%)	56,076 (100.0%)	56,037 (99.9%)	22,556 (40.2%)	9722 (17.3%)	4677 (8.3%)	2394 (4.3%)
	Males	Median (IQR)	33.49 (31.27; 35.67)	29.14 (26.80; 31.64)	29.33 (26.77; 31.87)	29.71 (27.16; 32.36)	29.79 (27.40; 32.39)	30.05 (27.62; 32.57)
		*N* (%)	13,530 (99.9%)	13,521 (99.9%)	5080 (37.5%)	2137 (15.8%)	1023 (7.6%)	510 (3.8%)
	Females	Median (IQR)	44.27 (41.66; 46.86)	39.68 (36.73; 42.67)	39.76 (36.68; 42.92)	40.19 (36.98; 43.14)	40.30 (37.15; 43.35)	40.52 (37.25; 43.56)
		*N* (%)	42,546 (100.0%)	42,516 (99.9%)	17,476 (41.1%)	7585 (17.8%)	3654 (8.6%)	1884 (4.4%)

Values are median (interquartile range). Sample sizes vary across variables and time points according to data availability and are reported for each analysis. Percentages indicate the proportion of the corresponding baseline group with available data at each time point. Values at each time point represent cross-sectional summaries of participants with available data at that assessment; therefore, differences between successive group medians should not be interpreted as within-person changes. BMI, body mass index.

## Data Availability

The data underlying this article are not publicly available because they contain health-related participant information. Deidentified data may be made available upon reasonable request to the corresponding author, subject to applicable French regulations and approval by the data holder.

## Supplementary Materials

The following supporting information can be downloaded at https://www.mdpi.com/article/10.3390/nu18203364/s1, Supplementary Methods: Sensitivity analysis for the availability of 4-year BMI; Table S1: Baseline characteristics of participants included and non-included in the analytical cohort; Table S2: Recruitment by calendar year and availability of 4-year follow-up measurements; Table S3: Absolute and relative changes in anthropometric characteristics from baseline to the end of the weight-loss phase; Table S4: Transitions between baseline and end-of-weight-loss BMI categories; Table S5: Adjusted changes in anthropometric characteristics from baseline to the end of the weight-loss phase and through 4-year follow-up; Table S6: Baseline and end-of-weight-loss characteristics according to availability of 4-year follow-up data; Table S7: Absolute and relative changes in anthropometric characteristics among participants with 4-year follow-up data; Table S8: Availability of 4-year BMI and inverse probability of observation weighting; Figure S1: Study flowchart; Figure S2: Changes in waist-to-height ratio and relative fat mass during the program; Figure S3: Transitions in BMI categories from baseline to the end of the weight-loss phase.
