# Supplementary material for: Weight Loss and Long-Term Weight Trajectories After a Structured Nutritional and Behavioral Intervention: A Large Real-World Cohort Study
Source: Nutrients. 2026 Oct 14;18(20):3364. doi: 10.3390/nu18203364 (PMC13708701; doi:10.3390/nu18203364)
Supplement: Supplementary file 1 [file nutrients-18-03364-s001.zip › nutrients-4589604-supplementary.pdf]

SUPPLEMENTARY MATERIALS

Weight Loss and Long-term Weight Trajectories After a Structured Nutritional and Behavioral Intervention: A Large Real-World Cohort Study

Supplementary Methods: Sensitivity analysis for the availability of 4-year BMI

Table S1. Baseline characteristics of participants included and non-included in the analytical cohort.

Table S2. Recruitment by calendar year and availability of 4-year follow-up measurements.

Table S3. Absolute and relative changes in anthropometric characteristics from baseline to the end of the weight-loss phase.

Table S4. Transitions between baseline and end-of-weight-loss BMI categories.

Table S5. Adjusted changes in anthropometric characteristics from baseline to the end of the weight-loss phase and through 4-year follow-up.

Table S6. Baseline and end-of-weight-loss characteristics according to availability of 4-year follow-up data.

Table S7. Absolute and relative changes in anthropometric characteristics among participants with 4-year follow-up data.

Table S8. Availability of 4-year BMI and inverse probability of observation weighting.

Figure S1. Study flowchart.

Figure S2. Changes in waist-to-height ratio and relative fat mass during the program.

Figure S3. Transitions in BMI categories from baseline to the end of the weight-loss phase.

Supplementary Methods: Sensitivity analysis for the availability of 4-year BMI

Participants were eligible for this analysis if they had baseline and end-of-weight-loss-phase (EWL) BMI measurements, baseline age and sex, an EWL date, and an opportunity to enter the predefined year-4 measurement window (days 1369–1825 after EWL) by the administrative data cutoff of December 31, 2025. An observed year-4 BMI was defined as an available BMI measurement selected within this window according to the study's time-point selection rules. Each participant contributed at most one year-4 measurement.

This eligibility definition differs from the nominal 4-year eligibility reported in the main analysis (N = 27,391), which required participants to have reached day 1460 after the end of the weight-loss phase. Because the predefined year-4 measurement window began at day 1369, participants could be eligible for the sensitivity analysis before reaching the nominal 4-year date, resulting in a larger eligible population (N = 29,190).

The probability of an observed year-4 BMI was estimated using logistic regression in the eligible population. Predictors were baseline age, sex and BMI; EWL BMI and calendar year; the most recent available BMI at EWL, year 1 or year 2; indicators of BMI availability at years 1 and 2; and the proportion of the year-4 window available before the administrative cutoff. Year-3 measurement availability was excluded from this model because the predefined year-3 and year-4 windows overlap.

For participants with an observed year-4 BMI, the stabilized inverse probability of observation weight was the overall proportion with an observed year-4 BMI divided by the participant's predicted probability of observation. We examined the distribution of predicted probabilities and weights and calculated the effective sample size as \((\sum w_i)^2/\sum w_i^2\). The primary weighted analysis truncated weights at their 1st and 99th percentiles; an analysis using untruncated weights assessed sensitivity to this choice. Mean individual BMI changes from baseline to year 4 were compared across the unweighted and weighted analyses. This approach assumes that, conditional on the variables in the observation model, availability of the year-4 measurement does not depend on the unobserved year-4 BMI, and that eligible participants have a positive probability of observation.

Table S1. Baseline characteristics of participants included and non-included in the analytical cohort.

	Not included N = 63,145	Included N = 56,093	Absolute SMD
BMI, kg/m^2	33.1 (29.8; 37.0)	31.9 (28.9; 35.6)	0.207
Weight, kg	90.8 (79.9; 104.2)	87.9 (77.6; 100.4)	0.171
Obesity (BMI ≥30 kg/m^2)	46,444 (73.6%)	37,265 (66.4%)	0.156
Body water, %	42.1(38.4; 45.2)	42.9 (39.6; 45.7)	0.151
Waist-to-height ratio	0.649 (0.598; 0.706)	0.636 (0.589; 0.691)	0.138
Age, y	53.0 (42.0; 62.0)	54.0 (45.0; 63.0)	0.137
Waist circumference, cm	108.0 (99.0; 118.0)	106.0 (97.0; 115.0)	0.134
Fat mass, %	41.2 (37.2; 45.4)	40.4 (36.6; 44.3)	0.120
Relative fat mass, %	43.4 (38.5; 46.6)	42.7 (37.6; 46.0)	0.116
Menopause among females	23,591 (48.3%)	22,994 (54.0%)	0.116
Current smoker	8,229 (13.0%)	5,577 (9.9%)	0.097
Osteoarthritis	18,802 (29.8%)	18,260 (32.6%)	0.060
Female sex	48,891 (77.4%)	42,556 (75.9%)	0.037
Sedentary lifestyle	21,796 (34.5%)	18,417 (32.8%)	0.036
Lean mass, %	29.1 (26.6; 32.2)	29.2 (26.9; 32.4)	0.024
Sleep apnea	11,136 (17.6%)	9,414 (16.8%)	0.023
Diastolic blood pressure, mmHg	85 (78; 92)	85 (78; 92)	0.020
MASH	2,975 (4.7 %)	2,548 (4.5%)	0.008
Systolic blood pressure, mmHg	130 (118; 142)	130 (120; 144)	0.006

Values are presented as median (interquartile range) for continuous variables and N (%) for categorical variables. The reference population comprised 119,238 participants who met the anthropometric eligibility criteria for the weight-loss phase. Participants who did not meet these criteria (N = 7,421) were not included in this comparison. Of the 119,238 participants, 56,093 were included in the analytical cohort, whereas 63,145 were not included because they had fewer than three measurements during the first 3 months (N = 25,578) or no measurement documenting entry into the weight-maintenance phase (N = 37,567). Percentages were calculated among participants with available data; menopause was assessed among females only. SMDs were calculated using pooled standard deviations for continuous variables and pooled variances of proportions for binary variables. Rows are ranked by decreasing absolute SMD. An absolute SMD ≥ 0.10 was considered indicative of a potentially meaningful difference between groups. SMD, standardized mean difference.

Table S2. Recruitment by calendar year and availability of 4-year follow-up measurements.

Year	Participants entry, N (%)	Participants EWL year, N	Nominally eligible for Y4, N	Y4 measurement among eligible, N (%)
**2013**	1,355 (2.4)	800	800	80 (10.0)
**2014**	1,702 (3.0)	1,576	1,576	207 (13.1)
**2015**	1,927 (3.4)	1,731	1,731	202 (11.7)
**2016**	2,249 (4.0)	1,969	1,969	191 (9.7)
**2017**	2,950 (5.3)	2,658	2,658	207 (7.8)
**2018**	3,780 (6.7)	3,354	3,354	224 (6.7)
**2019**	4,731 (8.4)	4,462	4,462	299 (6.7)
**2020**	4,637 (8.3)	4,096	4,096	369 (9.0)
**2021**	7,151 (12.7)	6,745	6,745	538 (8.0)
**2022**	6,350 (11.3)	6,562	0	—
**2023**	6,971 (12.4)	6,734	0	—
**2024**	7,353 (13.1)	7,244	0	—
**2025**	4,937 (8.8)	8,162	0	—
**Total**	**56,093**	**56,093**	**27,391**	**2,317 (8.5)**

Participants entering the program are classified according to the calendar year of program entry. EWL year refers to the calendar year in which participants completed the weight-loss phase. Nominal eligibility for 4-year follow-up was defined as reaching day 1,460 after EWL by the administrative data cutoff of December 31, 2025. Y4 measurement indicates the number and percentage of nominally eligible participants with at least one measurement within the prespecified year-4 window (days 1,369–1,825 after EWL). Percentages in the “Participants entry” column are calculated relative to the full analytical cohort (N = 56,093), whereas percentages in the “Y4 measurement among eligible” column are calculated relative to the number of nominally eligible participants. EWL, end of the weight-loss phase; Y4, year 4.

Table S3. Absolute and relative changes in anthropometric characteristics from baseline to the end of the weight-loss phase.

	All		Males		Females	
**Characteristic**	**Absolute change**	**Relative change (%)**	**Absolute change**	**Relative change (%)**	**Absolute change**	**Relative change (%)**
Weight, kg	-11.0 (-15.5; -7.8) (N = 56,093)	-12.7 (-16.7; -9.3)	-12.6 (-17.7; -9.0) (N = 13,537)	-12.3 (-16.2; -9.2)	-10.6 (-14.8; -7.4) (N = 42,556)	-12.8 (-16.8; -9.3)
Waist circumference, cm	-12.0 (-17.0; -9.0) (N = 56,029)	-11.8 (-15.4; -8.6)	-13.0 (-18.0; -10.0) (N = 13,517)	-11.4 (-14.9; -8.5)	-12.0 (-16.0; -9.0) (N = 42,512)	-11.8 (-15.5; -8.6)
Fat mass, %	-4.1 (-6.2; -2.5) (N = 49,671)	-10.3 (-15.4; -6.3)	-4.1 (-6.5; -2.6) (N = 12,079)	-11.8 (-18.6; -7.5)	-4.1 (-6.1; -2.5) (N = 37,592)	-9.8 (-14.6; -6.0)
Lean mass, %	1.3 (0.6; 2.4) (N = 49,632)	4.5 (2.1; 8.1)	2.1 (1.2; 3.3) (N = 12,058)	6.1 (3.6; 10.1)	1.1 (0.5; 2.0) (N = 37,574)	4.0 (1.8; 7.3)
Waist-to-height ratio	-0.075 (-0.101; -0.053) (N = 56,029)	-11.8 (-15.4; -8.6)	-0.075 (-0.100; -0.055) (N = 13,517)	-11.4 (-14.9; -8.5)	-0.075 (-0.101; -0.053) (N = 42,512)	-11.8 (-15.5; -8.6)
Relative fat mass, %	-4.2 (-5.7; -3.0) (N = 56,029)	-10.2 (-13.9; -7.1)	-4.0 (-5.3; -2.9) (N = 13,517)	-12.0 (-15.9; -8.6)	-4.3 (-5.8; -3.0) (N = 42,512)	-9.7 (-13.2; -6.8)

Values are median (interquartile range). N is shown below the absolute change estimate for each group. Analyses included participants with available measurements at baseline and EWL for the characteristic considered; therefore, N may vary across characteristics. Absolute change was calculated as EWL minus baseline, and relative change as 100 x (EWL - baseline)/baseline. Negative values indicate a decrease and positive values an increase. EWL, end of the weight-loss phase.

Table S4. Transitions between baseline and end-of-weight-loss BMI categories.

Baseline BMI categories	End of weight-loss BMI categories				Total
	18.5 to <25 kg/m²	25 to <30 kg/m²	30 to <35 kg/m²	≥35 kg/m²	
25 to <30 kg/m²	10,965 (58.2%)	7,850 (41.7%)	12 (0.1%)	1 (0.0%)	18,828
30 to <35 kg/m²	1,408 (6.6%)	16,381 (77.3%)	3,395 (16.0%)	7 (0.0%)	21,191
≥35 kg/m²	117 (0.7%)	3,332 (20.7%)	8,559 (53.2%)	4,066 (25.3%)	16,074
**Total**	12,490 (22.2%)	27,563 (49.1%)	11,966 (21.3%)	4,074 (7.3%)	56,093

Values are presented as numbers of participants. Rows represent baseline BMI categories and columns represent BMI categories at the end of the weight-loss phase. Within each baseline BMI category, percentages are calculated using the corresponding row total as the denominator and therefore indicate the proportion of participants transitioning to each BMI category at the end of the weight-loss phase. In the Total row, percentages are calculated using the overall sample (N = 56,093) as the denominator and indicate the distribution of BMI categories at the end of the weight-loss phase in the overall cohort. BMI, body mass index.

Table S5. Adjusted changes in anthropometric characteristics from baseline to the end of the weight-loss phase and through 4-year follow-up.

			Baseline to EWL		EWL to 4 years		Baseline to 4 years		
	Participants, N	Measurements, N	Adjusted Mean Change (95% CI)	P value	Adjusted Mean Change (95% CI)	P value	Adjusted Mean Change (95% CI)	P value	Overall P value for time
BMI, kg/m^2	56,093	95,511	-4.5 (-4.5 to -4.4)	<0.001	2.0 (1.9 to 2.0)	<0.001	-2.5 (-2.6 to -2.4)	<0.001	<0.001
Weight, kg	56,093	95,511	-12.3 (-12.4 to -12.3)	<0.001	5.4 (5.2 to 5.5)	<0.001	-7.0 (-7.1 to -6.8)	<0.001	<0.001
Waist circumference, cm	56,040	95,374	-13.2 (-13.3 to -13.2)	<0.001	4.0 (3.7 to 4.2)	<0.001	-9.3 (-9.5 to -9.0)	<0.001	<0.001
Fat mass, %	50,371	83,411	-4.6 (-4.7 to -4.6)	<0.001	2.7 (2.6 to 2.9)	<0.001	-1.9 (-2.0 to -1.7)	<0.001	<0.001
Lean mass, %	50,327	83,318	1.7 (1.7 to 1.8)	<0.001	-0.8 (-0.9 to -0.7)	<0.001	0.9 (0.8 to 1.1)	<0.001	<0.001
Waist-to-height ratio	56,040	95,374	-0.080 (-0.080 to -0.079)	<0.001	0.024 (0.023 to 0.025)	<0.001	-0.056 (-0.057 to -0.054)	<0.001	<0.001
Relative fat mass, %	56,040	95,374	-4.5 (-4.5 to -4.4)	<0.001	1.5 (1.4 to 1.6)	<0.001	-2.9 (-3.0 to -2.9)	<0.001	<0.001

Values are adjusted mean changes (95% CI) from separate linear mixed-effects models including all available repeated measurements. Models included time as a categorical fixed effect, a patient-level random intercept, and adjustment for baseline age, sex, and the baseline value of the anthropometric characteristic. The EWL-to-4-year estimate represents the difference between adjusted changes at 4 years and the end of weight-loss phase. EWL, end of the weight-loss phase; CI, confidence interval.

Table S6. Baseline and end-of-weight-loss characteristics according to availability of 4-year follow-up data.

	No 4-year follow-up N = 53,698	4-year follow-up available N = 2,395	Absolute SMD
**Baseline characteristics**			
Age, y	54.0 (45.0; 63.0)	56.0 (47.0; 64.0)	0.089
Female sex	40,672 (75.7%)	1,884 (78.7%)	0.070
Weight, kg	88.0 (77.7; 100.5)	85.3 (76.0; 98.3)	0.119
BMI, kg/m^2	31.9 (28.9; 35.6)	31.3 (28.6; 35.1)	0.085
Obesity (BMI ≥30 kg/m^2)	35,773 (66.6%)	1,492 (62.3%)	0.090
Waist circumference, cm	106.0 (97.0; 115.0)	104.0 (96.0; 114.0)	0.077
Fat mass, %	40.4 (36.6; 44.4)	40.1 (37.0; 43.7)	0.034
Lean mass, %	29.2 (26.9; 32.5)	29.1 (27.3; 32.1)	0.035
Waist-to-height ratio	0.637 (0.589; 0.692)	0.633 (0.585; 0.688)	0.041
Relative fat mass, %	42.7 (37.6; 46.0)	42.7 (38.4; 45.8)	0.033
**Characteristics at EWL**			
Weight, kg	75.9 (67.5; 86.9)	71.5 (64.9; 81.6)	0.283
BMI, kg/m^2	27.5 (25.2; 30.5)	26.4 (24.4; 29.1)	0.292
Obesity (BMI ≥30 kg/m^2)	15,572 (29.0%)	468 (19.5%)	0.222
Waist circumference, cm	93.0 (85.0; 101.0)	90.0 (83.0; 98.0)	0.246
Fat mass, %	36.1 (32.1; 40.1)	35.5 (32.3; 39.0)	0.059
Lean mass, %	30.8 (28.2; 34.8)	30.4 (28.2; 33.8)	0.021
Waist-to-height ratio	0.558 (0.516; 0.606)	0.543 (0.506; 0.588)	0.216
Relative fat mass, %	37.9 (32.9; 41.7)	37.4 (32.8; 40.8)	0.087
**Changes from baseline to EWL**			
Absolute weight change, kg	-11.0 (-15.4; -7.7)	-12.5 (-17.5; -9.1)	0.268
Relative weight change, %	-12.6 (-16.5; -9.2)	-14.8 (-18.9; -11.3)	0.386
Absolute waist change, cm	-12.0 (-17.0; -9.0)	-14.0 (-19.0; -10.0)	0.267
Relative waist change, %	-11.7 (-15.3; -8.5)	-13.4 (-17.2; -10.0)	0.322
Absolute fat mass change, percentage points	-4.0 (-6.2; -2.5)	-4.3 (-6.2; -2.9)	0.018
Absolute lean mass change, percentage points	1.3 (0.6; 2.4)	1.3 (0.6; 2.1)	0.102

Values are presented as median (interquartile range) for continuous variables and number (percentage) for categorical variables. Changes were calculated as the value at the end of the weight-loss phase minus the baseline value; relative changes were calculated as 100 × (EWL value − baseline value)/baseline value. Changes in fat mass and lean mass are expressed in percentage points. Absolute SMDs were used to assess differences between participants with and without available 4-year follow-up data; values ≥ 0.10 were considered indicative of a potentially meaningful imbalance. BMI, body mass index; EWL, end of the weight-loss phase; SMD, standardized mean difference.

Table S7. Absolute and relative changes in anthropometric characteristics among participants with 4-year follow-up data.

		From baseline to EWL			From EWL to 4 years			From baseline to 4 years		
	N	Absolute change (units as shown)	Relative change (%)	SRM	Absolute change (units as shown)	Relative change (%)	SRM	Absolute change (units as shown)	Relative change (%)	SRM
**Weight, kg**										
All	2,395	-12.5 (-17.5; -9.1)	-14.8 (-18.9; -11.3)	1.9	4.7 (0.9; 8.8)	6.5 (1.3; 12.1)	0.7	-7.8 (-13.1; -3.7)	-9.1 (-14.5; -4.4)	1.0
Males	511	-15.0 (-20.1; -10.6)	-14.5 (-18.4; -11.1)	2.1	5.0 (0.9; 9.6)	5.8 (1.0; 11.2)	0.7	-9.5 (-14.7; -4.7)	-9.3 (-14.0; -5.0)	1.1
Females	1,884	-12.0 (-16.7; -8.7)	-14.9 (-19.0; -11.4)	1.9	4.6 (0.9; 8.5)	6.8 (1.3; 12.5)	0.7	-7.2 (-12.4; -3.3)	-9.0 (-14.6; -4.1)	1.0
**Waist circumference, cm**										
All	2,393	-14.0 (-19.0; -10.0)	-13.4 (-17.2; -10.0)	2.2	4.0 (-1.0; 9.0)	3.9 (-1.1; 9.4)	0.5	-10.0 (-16.0; -5.0)	-9.8 (-15.1; -4.9)	1.2
Males	510	-15.0 (-20.0; -11.0)	-13.1 (-16.8; -9.8)	2.3	5.0 (0.0; 10.0)	4.5 (0.0; 9.9)	0.6	-10.0 (-16.0; -5.0)	-8.9 (-13.5; -4.3)	1.2
Females	1,883	-13.0 (-18.0; -10.0)	-13.5 (-17.3; -10.0)	2.1	3.0 (-1.0; 8.0)	3.8 (-1.1; 9.1)	0.5	-10.0 (-16.0; -5.0)	-10.1 (-15.5; -5.0)	1.2
**Fat mass, %**										
All	1,940	-4.3 (-6.1; -2.8)	-10.9 (-15.1; -7.3)	1.3	2.4 (0.3; 4.8)	6.5 (0.7; 13.7)	0.5	-1.9 (-4.6; 0.4)	-4.6 (-11.2; 1.0)	0.4
Males	408	-4.4 (-6.2; -3.2)	-12.5 (-17.5; -9.3)	1.1	1.9 (-0.9; 4.3)	6.0 (-2.9; 14.2)	0.3	-2.8 (-6.1; -0.5)	-8.0 (-16.1; -1.5)	0.5
Females	1,532	-4.2 (-6.1; -2.7)	-10.4 (-14.6; -6.7)	1.4	2.5 (0.5; 4.9)	6.6 (1.3; 13.7)	0.6	-1.7 (-4.1; 0.6)	-4.0 (-10.0; 1.3)	0.4
**Lean mass, %**										
All	1,938	1.2 (0.6; 2.1)	4.1 (2.1; 6.9)	0.4	-1.0 (-2.1; -0.2)	-3.3 (-6.7; -0.6)	0.1	0.1 (-0.7; 1.3)	0.4 (-2.4; 4.3)	0.1
Males	407	2.1 (1.4; 3.1)	6.0 (4.0; 8.8)	0.6	-1.4 (-2.9; -0.2)	-4.0 (-7.8; -0.6)	0.1	0.6 (-0.7; 2.2)	1.7 (-1.8; 6.3)	0.2
Females	1,531	1.0 (0.5; 1.8)	3.6 (1.8; 6.3)	0.4	-0.9 (-1.9; -0.1)	-3.2 (-6.5; -0.6)	0.1	0.0 (-0.7; 1.1)	0.0 (-2.5; 3.8)	0.1
**Waist-to-height ratio**										
All	2,393	-0.083 (-0.113; -0.061)	-13.4 (-17.2; -10.0)	2.2	0.022 (-0.006; 0.051)	3.9 (-1.1; 9.4)	0.5	-0.062 (-0.096; -0.030)	-9.8 (-15.1; -4.9)	1.2
Males	510	-0.086 (-0.114; -0.063)	-13.1 (-16.8; -9.8)	2.3	0.027 (0.000; 0.056)	4.5 (0.0; 9.9)	0.6	-0.058 (-0.090; -0.028)	-8.9 (-13.5; -4.3)	1.2
Females	1,883	-0.083 (-0.112; -0.061)	-13.5 (-17.3; -10.0)	2.1	0.019 (-0.006; 0.050)	3.8 (-1.1; 9.1)	0.5	-0.063 (-0.098; -0.031)	-10.1 (-15.5; -5.0)	1.2
**Relative fat mass, %**										
All	2,393	-4.9 (-6.4; -3.6)	-11.8 (-15.7; -8.7)	2.3	1.4 (-0.4; 3.1)	3.7 (-1.0; 8.7)	0.5	-3.4 (-5.6; -1.6)	-8.5 (-13.3; -3.8)	1.2
Males	510	-4.7 (-6.0; -3.5)	-13.9 (-18.1; -10.4)	2.4	1.5 (0.0; 3.3)	5.2 (0.0; 11.8)	0.6	-3.0 (-4.8; -1.4)	-9.2 (-14.1; -4.0)	1.2
Females	1,883	-5.0 (-6.6; -3.6)	-11.3 (-14.9; -8.2)	2.3	1.3 (-0.4; 3.1)	3.4 (-1.0; 8.1)	0.5	-3.6 (-5.8; -1.7)	-8.2 (-13.1; -3.8)	1.2

Values are median (interquartile range). Analyses included participants with available measurements at baseline, end of weight-loss phase (EWL), and 4-year follow-up for the characteristic considered; therefore, N may vary across characteristics. Absolute and relative changes represent within-person changes. Absolute change was calculated as the later minus the earlier value, and relative change as 100 × (later value − earlier value)/earlier value. Positive values indicate an increase and negative values a decrease. The SRM was calculated as the absolute mean individual change divided by the standard deviation of individual changes, separately for the overall population, males, and females. For example, an SRM of 1.9 indicates a mean change corresponding to 1.9 standard deviations of individual changes. SRMs were interpreted as negligible (< 0.20), small (0.20–0.49), moderate (0.50–0.79), or large (≥ 0.80). EWL, end of the weight-loss phase; SRM, standardized response mean.

Table S8. Availability of 4-year BMI and inverse probability of observation weighting.

Characteristic	Value
**Analysis population**	
Eligible participants	29,190
Year-4 BMI observed	2,395 (8.20%)
Year-4 BMI unavailable	26,795 (91.80%)
Observed year-4 measurement within days 1369–1825	2,395/2,395 (100%)
**Predicted probability of year-4 observation among observed participants**	
Median	0.301
1st–99th percentiles	0.010–0.549
**Stabilized weights among observed participants, before truncation**	
Median	0.273
1st–99th percentiles	0.150–8.039
Maximum	13.196

Mean individual BMI change from baseline to year 4		
**Analysis**	**Estimate (kg/m^2)**	**95% CI**
Unweighted	−3.25	−3.37 to −3.12
Inverse probability of observation weighted; weights truncated at the 1st and 99th percentiles	−1.92	−2.18 to −1.66
Inverse probability of observation weighted; untruncated weights	−1.89	−2.16 to −1.62

Eligible participants were those with baseline and end-of-weight-loss-phase (EWL) BMI measurements, baseline age and sex, an EWL date, and an opportunity to enter the predefined year-4 measurement window (days 1369–1825 after EWL) before the administrative data cutoff of December 31, 2025. Stabilized inverse probability of observation weights were calculated as described in the Supplementary Methods. The primary weighted analysis used weights truncated at the 1st and 99th percentiles; estimates using untruncated weights are also shown. BMI, body mass index; CI, confidence interval; EWL, end of the weight-loss phase.

Figure S1. Study flowchart.

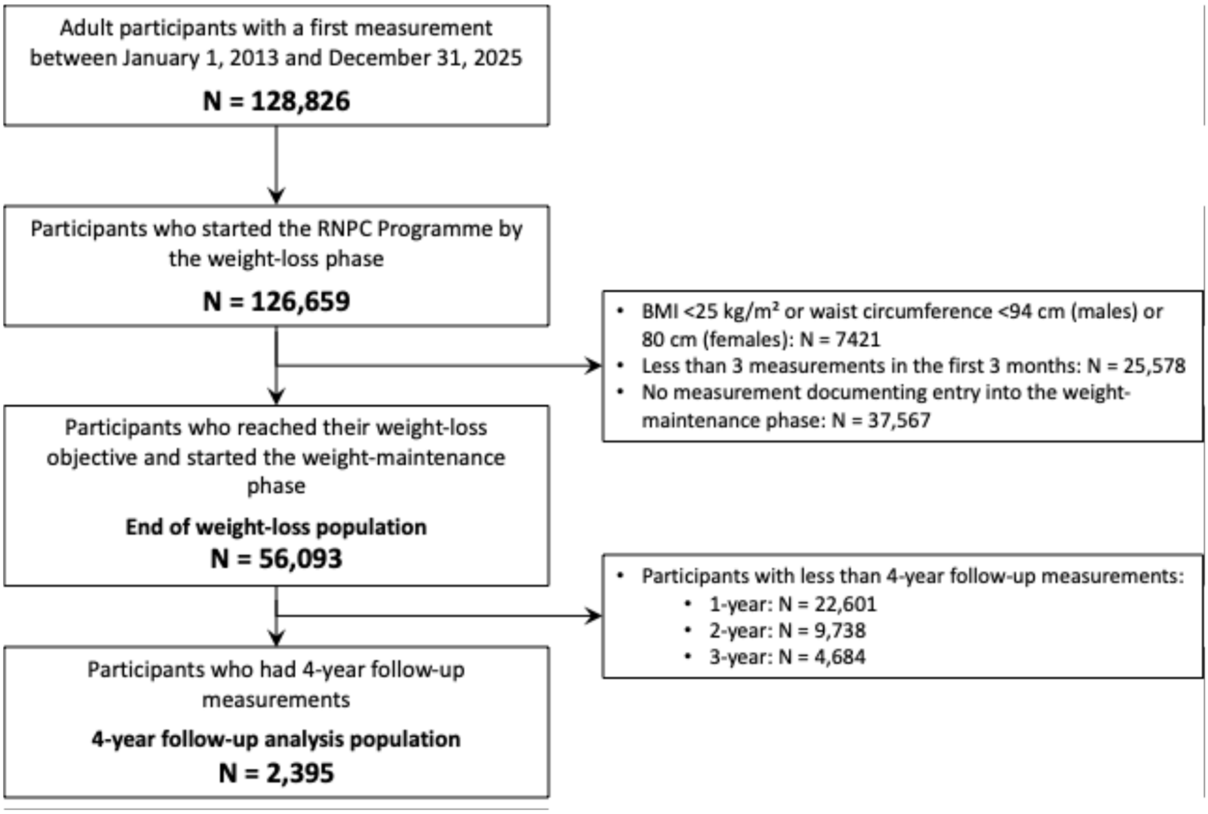

Figure S2. Changes in waist-to-height ratio and relative fat mass during the program.

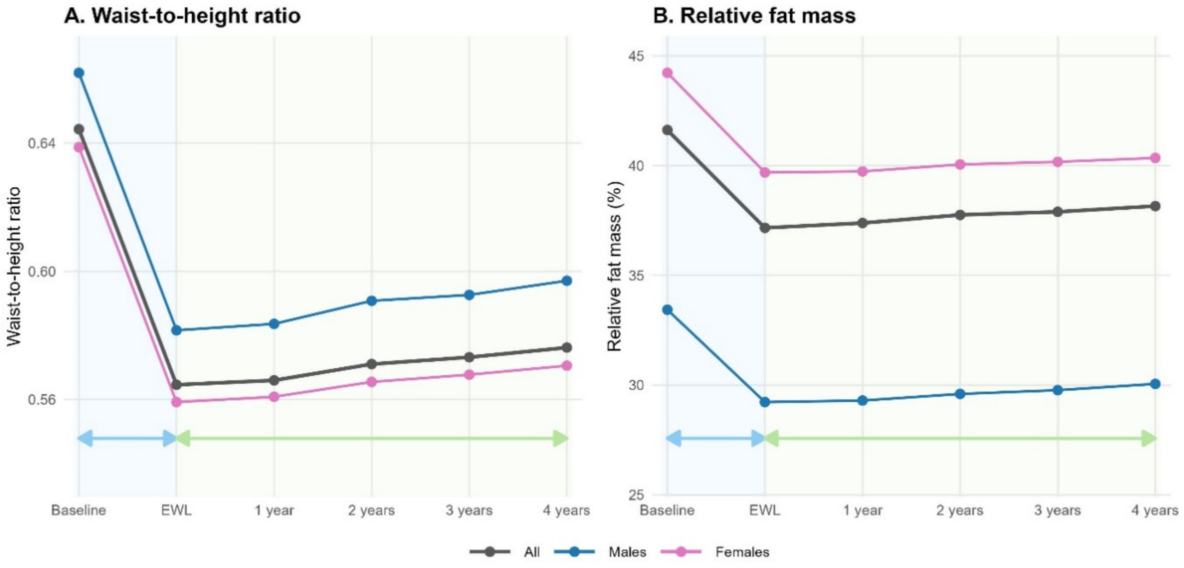

Figure S3. Transitions in BMI categories from baseline to the end of the weight-loss phase.

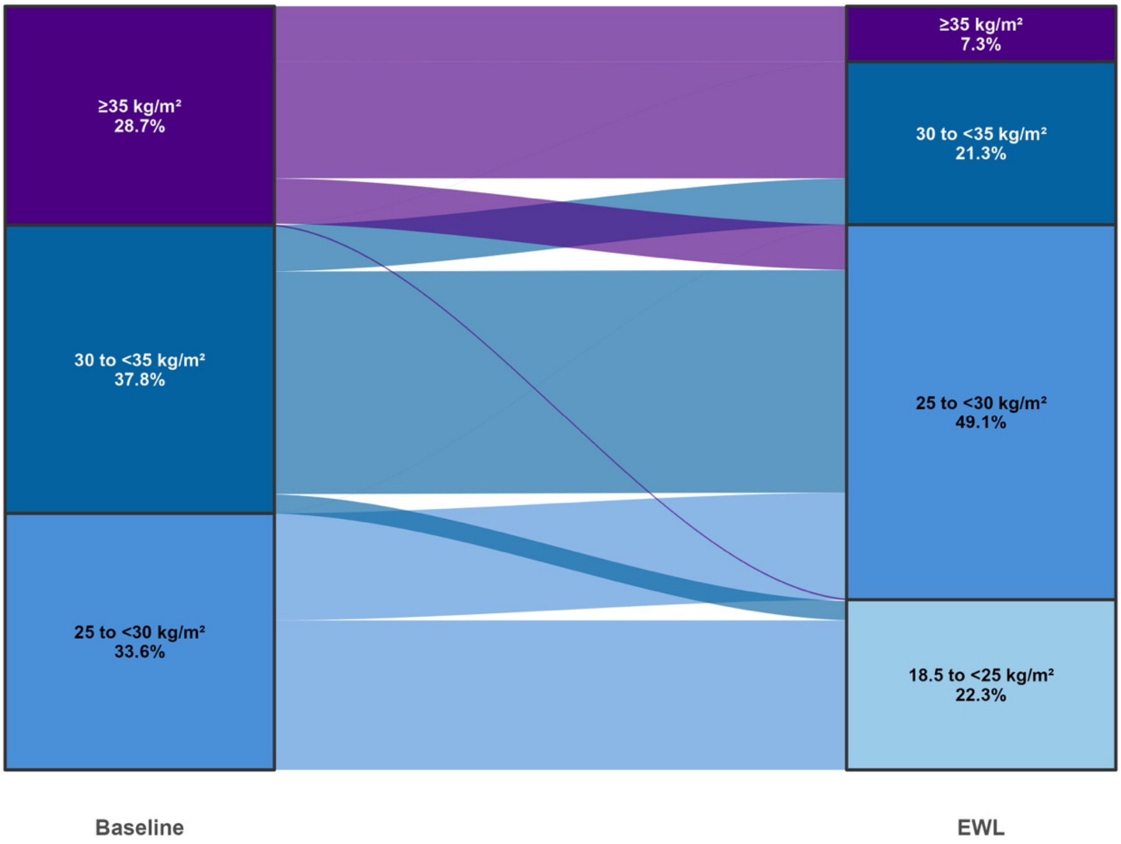

Sankey/alluvial plot illustrating transitions between BMI categories from baseline to the end of the weight-loss phase (EWL) (N = 56,093). The width of the flows is proportional to the number of participants moving between BMI categories. BMI, body mass index.
